# Bilateral frontal sinus mucocele

**DOI:** 10.1016/S1808-8694(15)30981-2

**Published:** 2015-10-19

**Authors:** Flavio Akira Sakae, Bernardo Cunha Araújo Filho, Marcus Lessa, Richard Lois Voegels, Ossamu Butugan

**Affiliations:** a Post-graduate student and physician of the Otorhinolaryngological Clinical Division of the USP Medical College; b Post-graduate student and physician of the USP Medical College; c Collaborating physician of the Otorhinolaryngology Department of the Federal Universityof Bahia; d Physician, professor of the Otorhinolaryngological Clinical Division of the USP Medical College, associate professor of the Otorhinolaryngological Clinical Division of the USP Medical College; e Professor of the Otorhinolaryngological Clinical Division of the USP Medical College, associate professor of the Otorhinolaryngological Clinical Division of the USP Medical College. Clinical Hospital of the Sao Paulo University Medical College

**Keywords:** endoscopic sinus surgery, frontal sinus, mucocele

## INTRODUCTION

Mucoceles are benign lesions, covered by pseudostratified epithelium, that affect paranasal sinuses. Most of them occur in the frontal sinus (60%). Bilateral involvement is extremely rare^1^.

## CASE REPORT

**Figure 1:**
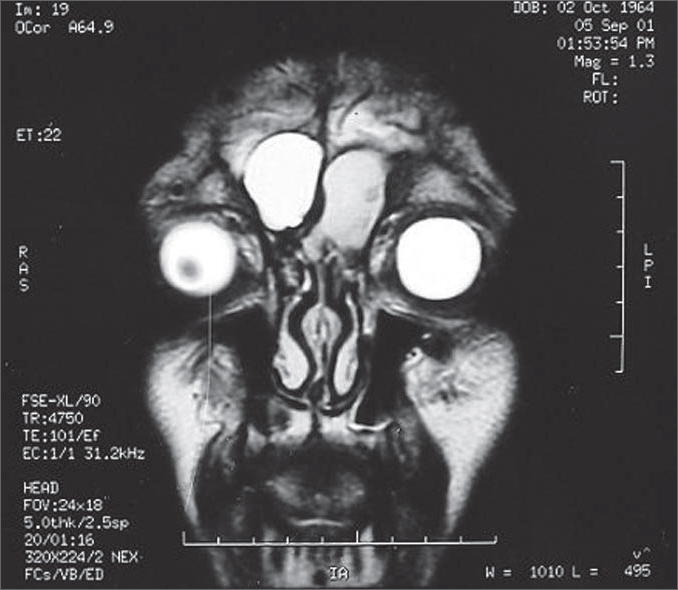
Magnetic resonance image of paranasal sinuses, coronal section, T2. Bilateral Frontal Mucocele.

A male patient aged 37 years with a history of cranioencephalic trauma 21 years ago, reports a left frontal tumor beginning 4 months ago. Magnetic resonance imaging revealed a lesion suggesting mucocele (Figure 1). The patient underwent endoscopic bilateral frontal ethmoid sinusectomy. The option was made for external left frontal ethmoidectomy due to intense bone sclerosis in the frontal recess. The intersinusal septum was intact and was opened during the procedure. At 5 months follow-up there is no evidence of mucocele recurrence.

## DISCUSSION

Mucoceles develop following obstruction of the drainage ostia, growing slowly within the sinuses, eventually eroding adjacent bone structures^1^. The etiology includes inflammatory processes, neoplasms, post-operative complications and post-traumatic sequelae^2^.

Mucoceles are mostly located in the frontal sinus, although bilateral involvement is extremely rare^1^, ^3^. In literature only two such cases have been reported^2^, ^4^.

The diagnosis is made based on the clinical history, physical examination and radiologic imaging. Symptoms vary in frontal ethmoid mucocele from absence of symptoms to incapacitating pain, headache and visual disturbances^3^.

Computed tomography shows an isodense homogeneous image, with loss of the normal sinus contour that is not enhanced with contrast if not infected^3^.

Magnetic resonance imaging is indicated if there is any doubt in diagnosis. The typical finding is a T1 hyposignal area T1 and a T2 hypersignal area, however any combination of signal intensities may be found depending on the presence of blood particles or the degree of hydration of the content^5^.

Currently ample marsupialization of the mucocele and simple drainage of the sinus by endoscope has been done with excellent surgical results. The advantages include low morbidity, low risk of complication and a rare recurrence rate^6^. The surgeon should have deep knowledge of frontal recess anatomy to perform endoscopic treatment of this condition.

## FINAL COMMENTS

Endoscopic treatment seems to be the best treatment option. However, for technical reasons anatomical variants may preclude ample marsupialization done only endoscopically.
